# Are the Innovative Electronic Labels for Extra Virgin Olive Oil Sustainable, Traceable, and Accepted by Consumers?

**DOI:** 10.3390/foods8110529

**Published:** 2019-10-25

**Authors:** Simona Violino, Federico Pallottino, Giulio Sperandio, Simone Figorilli, Francesca Antonucci, Vanessa Ioannoni, Daniele Fappiano, Corrado Costa

**Affiliations:** 1 Consiglio per la ricerca in agricoltura e l’analisi dell’economia agraria (CREA) - Centro di ricerca Ingegneria e Trasformazioni agroalimentari, Via della Pascolare 16, 00015 Monterotondo (Rome), Italy; simonaviolino@hotmail.com (S.V.); federico.pallottino@crea.gov.it (F.P.); giulio.sperandio@crea.gov.it (G.S.); simone.figorilli@crea.gov.it (S.F.); francesca.antonucci@crea.gov.it (F.A.); 2 Istituto Nazionale di Statistica (ISTAT) - Direzione centrale per le statistiche sociali e il censimento della popolazione (DCSS) - Servizio registro della popolazione, statistiche demografiche e condizioni di vita (SSA), Viale Liegi 13, 00198 Rome, Italy; ioannoni@istat.it; 3 Maticom S.r.l.-Via Carlo Spinola 5, 00154 Rome, Italy; daniele.fappiano@gmail.com

**Keywords:** EVOO, smart tag, Internet of Food (IoF), food safety, customer, blockchain

## Abstract

Traceability is the ability to follow the displacement of food through its entire chain. Extra virgin olive oil (EVOO) represents Italian excellence, with consumers’ increased awareness for traceability. The aim of this work is to propose and analyze the economic sustainability and consumers’ preference of three technological systems supporting traceability: Near Field Communication (NFC) based; tamper-proof device plus Radio Frequency Identification (RFID) and app; QR code tag plus “scratch and win” system and blockchain. An anonymous questionnaire to Italian consumers (*n* = 1120) was made to acquire consumers’ acceptability of the systems and estimating their willingness to pay additional premium prices for these. An economic analysis estimated and compared the technology costs at different production levels. Results show that 94% of the consumer respondents are interested in the implementation of such technologies, and among them 45% chose QR-code protected by a “scratch-and-win” system with a blockchain infotracing-platform (QR-B). The consumers interested are willing to pay a mean premium price of 17.8% and economic analysis reported evidenced an incidence always lower than mid-/high-production levels. The success of the QR-B could be ascribed to different aspects: the cutting-edge fashion trend of blockchain in the food sector, the use of incentives, the easy-to-use QR-code, and the gamification strategy.

## 1. Introduction

Traceability is usually defined as the ability to follow the movement of a product through specific phases of production, processing, and distribution tracking the history, the origin of materials and parts, the processing, the distribution, and the position of the product [1]. Backward traceability (tracing) is defined as the process that allows to trace back a product and its transformations within crucial steps of the food chain, to identify a specific event or action [2]. Nowadays, one of the main problems in the food industry is to define tools to identify the origin of raw materials of food products in order to guarantee their traceability and tracing [3].

One of food products at the base of Mediterranean diet is extra virgin olive oil (EVOO); this foodstuff plays an important role in international food commerce. The Report of the Parliamentary Committee Inquiry on Counterfeiting signalized that authentic olive oil is under an increasing the risk of adulteration [4]. A traceability system could be a direct system able to fight counterfeit EVOO. Methods supporting EVOO traceability are continuously evolving to optimize the supply chain, increase the prevention of mislabeling in relation with geographical origin and olive cultivar, and assuring the presence of correct and additional information for the consumers [5]. Since the largest main traceability weakness is related to the certain attribution of the geographical origin, the European Commission, to safeguard the identification of extra virgin olive oil, applied two kinds of certification, namely Protected Designation of Origin (PDO) and Protected Geographical Indication (PGI) [6]. Italy is the second biggest olive oil producer in the world, after Spain, with an average production of 555,574 tons from 1993 to 2014 [7]. A total of 80% of Italian’s olive oil production is concentrated in the Southern regions. In Italy, according to Census data [8], counterfeiting is a growing phenomenon on par with a similar EU trend. As reported by numerous surveys, the continuous counterfeiting linked to “made in Italy” is leading to some new phenomena such as the so-called “Italian Sounding” [9], namely the imitative practice that causes damage to the image of an Italian product. Such a problem leads to increase safety requirements and consumer protection systems with regard to the oil quality and security from “field to fork”.

Generally, EVOO costs 4–5 times more than other vegetables oils. This is mainly imputable to the higher production costs as well as for its higher nutritional and organoleptic properties [10], therefore, the higher cost should be justified by higher quality guarantees. The implementation of a traceability system for EVOO represents a significant running cost for producers and processing companies. The price paid does include the resources to set-up (scale on the base of the production needs) and run the system. Such a cost should be in an acceptable range that would incentivize companies to adopt traceability systems in order to receive reasonable benefits with positive repercussions concerning information reliability, effectiveness, and productivity depending upon the organization [11]. In the competitive market, the increase of costs, as a result of traceability systems, can rebound in the consumer with little change in quantity demanded because of relatively lower food demand price elasticity compared to most industrial products [12]. However, in recent years consumers’ willingness to pay for food traceability has been given increasing attention due to the worldwide concern about food safety [12]. It is very important that companies, in balancing benefits and costs, understand consumers’ preferences about advantages, costs, and reliability of traceability systems. However, it is important that knowledge related to food quality, security, and certification is seen by consumers a crucial factor in terms of “willingness to pay” for a food product with implementation of traceability system [12,13]. Traceability will be increasingly integrated with smart sensing systems and consequently add data about product features, production methods, and ambient conditions (Internet of Food (IoF)) [14].

A process to keep records reporting the trail of an input from suppliers to customers is defined as an infotracing system [15]. At the end of the infotracing flow, consumers acquire information on the product by reading a physical label [16] or a digital application. Currently, it is possible to obtain information about traceability along the food supply chain using smart tags such as Radio Frequency Identification (RFID), Near Field Communication (NFC), and the most diffused barcodes. This last example is commonly present in the food market as 1D barcodes or 2D codes (i.e., Quick Response (QR) code) [17]. Other technologies use electronic tags. These tags store information in a digital format that can be read by a compatible scanner using small integrated circuits. The most used ones, with varying underlying communication technologies, are NFC/RFID. RFID tags report logistic advantages being automatable and readable within logistic platforms [17]. Many studies demonstrate, for food traceability purposes, RFID systems produce more advantages when compared to the barcode ones [18]. For example, Gandino et al. [19] proposed the RFID adoption for fruit warehouse traceability, remarking many benefits for the supply chain management, commodity value addition, and brand management. There are also visible or electronic markers (2D codes, NFC) with environmental sensing functions (functional ink, sensors, indicators) combined with software intelligence (user information, location, etc.) in order to provide context aware services to end users. In the study by Mainetti et al. [20] an NFC system was implemented to trace an IV range products supply chain in conjunction with a mobile app to allow the linking of plants and traceability information. These smart tags should increase the consumer trust towards food products (integrity, quality, safety, authenticity) and service providers (food value chains) [21]. Today, consumers are familiar with QR codes and NFC, but they are not willing to read them regularly using their mobile phones; they feel that they do not get any additional value from it. It is essential that the consumers trust the service and service provider [22,23].

Currently, these modern technologies are proposed for food traceability systems. Tarjan et al. [24], present a concept for fruit yogurts labeling based on QR codes, which enables traceability of products and provides additional information about the peculiarities of the product. Another example of smart technologies used for food traceability is NFC. In the work by Pigini et al. [25], they proposed a solution based on NFC tags to obtain information in the pig supply chain in order to inform the consumer.

The aim of this work is to explore the willingness of the consumers to accept and adopt EVOO electronic traceability systems and explore the economic sustainability their introduction and use in a stable market such as the EVOO one. To pursue this aim, we proposed a questionnaire to a limited number of consumers, proposing three alternative technologies for traceability (NFC, tamper-proof device protected by RFID sticker, and QR code protected by a “scratch and win” system and associated with blockchains), and analyzed the economic feasibility of the introduction of these technologies at three different production levels, representative of Italian EVOO production panorama. This work is organized into a Materials and methods, which illustrate the innovative electronic labels proposed, the questionnaire disseminated to consumers and private companies, and the economic sustainability analysis. The results and discussion section is composed by a section regarding the consumer interviews, another related to the stakeholder interviews, and the results of the economic analysis.

## 2. Materials and Methods

Extra virgin olive oil (EVOO) owes its quality to different factors such as cultivar typology, environmental conditions, and cultural practices. It is a product of proximity and high added value for the consumer. In fact, the best EVOO oils are PDO oils due to their genuine and specific organoleptic characteristics. As a consequence, consumers are increasingly oriented towards purchasing food products of a certified authenticity and geographical origin [26].

### 2.1. The Innovative Electronic Labels Proposed

Three different technologies for bottled EVOO traceability were proposed through a questionnaire to acquire consumers’ acceptability. Table 1 reports the different innovative electronic labels used for the questionnaire of this study.

The NFC tag is placed underneath the external label. Consumers activating the smartphone NFC antenna can read additional information regarding the product traceability and its production origin.

The Dispositivo Antimanomissione per Bottiglie (DAB), [27] is a patented tamper-proof device composed of a newly designed bottle cap, which externally covers the traditional one. A RFID [25] adhesive label, containing a unique code, is applied to close the two valves of the additional bottle cap. The code is sent to the on-line information system which unlocks the tamper-proof device, saves in memory the purchased products at the store, and generates a Unique Purchase Code (UPC). This can be read by the consumer with a specific mobile application (e.g., reading a barcode). UPC is afterwards interrogated by the application allowing to trace back the product data on traceability, those previously registered in the Certified Information System by mapping the territory.

Finally, the QR-B system is composed of a unique QR code tag printed on the external label and protected by a “scratch and win” system. The “scratch and win” system is a gamification method which protects the QR-code in order to allow the consumer who buys the product to obtain the reward. The infotracing platform [28] is implemented with blockchains [15,29]. Once the consumer purchases the product, scratching the label and framing the QR code with a smartphone, he receives, along with the product information, a digital token of €0.05, produced by the blockchain itself, that can be spent on the reference platform. The blockchain system allows a guarantee that the information inserted along the entire supply chain is tamper-proof. In this case, the innovation is represented not only by the introduction of blockchains, which could be implemented also in NFC and RFID (DAB) technologies, but also by the combined use of the “scratch and win” system protecting the consumer. The system includes standard motivational models and technological models within the reach of the consumer.

### 2.2. The Questionnaire: Consumer and Company Interviews

The questionnaire was launched through the CREA website, the FB CREA’s page, and the Agronotizie web magazine. It was disseminated through two different methodologies. Consumer interviews were carried out by Computer Assisted Web Interview (CAWI) on a self-compiled web-based platform and Pen-and-Paper Personal Interview (PAPI) face-to-face interview. The PAPI were conducted at a local fair in Grottaferrata (Central Italy) on 23–24 March 2019. The questionnaire filled in through CAWI included three short videos in Italian language illustrating the proposed technologies (DAB: https://www.youtube.com/watch?v=lEdwWGd2TdM, NFC: https://www.youtube.com/watch?v=Jtx97C2JOBs, QR-B: https://www.youtube.com/watch?v=szsbLFhPi1s). The self-compiled web-based platform was implemented in Microsoft Forms and remained active from 29 March to 30 May 2019.

Moreover, three extra virgin olive oil producers/suppliers were interviewed to evaluate a real degree of appreciation, by companies, for each of three systems proposed. Stakeholder interviews were carried out by Pen-and-Paper Personal Interview (PAPI) face-to-face interview. The companies were chosen to be representative for each one of the three different EVOO production levels (farm, consortium, and industrial).

The survey aimed, first, to reveal the interest of consumers in receiving additional information on the origin, traceability, and quality of extra virgin olive oil. On the basis of this preliminary information, the consumers were asked to express an opinion regarding the willingness to pay for an extra cost over the conventional selling price, the budget they would be willing to pay for that service, and which technology was the preferred among the three proposed. The questionnaire was divided into two sections: short socio-demographic questions and specific questions related to the research’s aim. Table 1 reports the questions proposed in questionnaire to unpaid consumers.

Socio-demographic questions (Q1–Q4; Table 2) regarded gender, age, geographical area of residence, and owning a university degree. Specific questions (Q5–Q9; Table 2) were inherent: eventual consumption of quality extra virgin olive oil, high quality extra virgin olive oil purchased on average (APO; Q6 Table 2), interest toward the traceability of extra virgin olive oil, willingness to pay for an additional cost paying for traceability technologies (WTP), acceptable additional cost to pay for these technologies (AAP; derived from Q8 Table 2). The core of the questionnaire was the question on the choice of one of the traceability technologies proposed. Consumers were asked to choose among the three technologies proposed (NFC, DAB, or QR-B; see Section 2.1).

Mean and standard deviation values of APO and AAP with respect to the categories gender, age class, geographical area, and university degree were reported. Moreover, a Normality test Shapiro–Wilk was applied to verify the normality of distribution of the APO and AAP answers where the null hypothesis (H_0_ normality) was confirmed for *p* value greater than 0.05. Afterwards, based on the results of the aforementioned test (Shapiro–Wilk Normality test always showed *p* values lower than 0.05) a non-parametric Kruskal–Wallis test (H_0_ equality of medians) was conducted in order to test the equality of the medians. Mann–Whitney pairwise comparisons (Bonferroni corrected) post hoc test was performed. Those tests were performed using the free software PAST (2.17c, Øyvind Hammer, Natural History Museum, University of Oslo, Oslo, Norway) [30]

Considering the consumers choice for the three proposed technologies, χ^2^ tests were performed with respect to the categories gender, age class, geographical area, and owning a university degree. The χ^2^ components statistics for goodness-of-fit tests was also calculated in order to observe case-specific significant differences [31].

### 2.3. Economic Analysis

The economic analysis was based on the estimation and comparison of costs linked to the implementation of the three different technologies for traceability of the EVOO production chain. The analysis considered the three systems previously aforementioned (Section 2.1; NFC, DAB, and QR-B). Different economic scenarios were proposed, based on three representative levels of EVOO production:

1. Farm level, production 10,000 L per year;
2. Consortium level, production 200,000 L per year;
3. Industrial level, production 30,000,000 L per year.

For each of these production levels, three different options were analyzed in relation to the types of bottles commonly used for packaging and distribution on the Italian market, distinguished based on the volume: (i) 1 L; (ii) 0.75 L; (iii) 0.25 L. In Table 3, the number of bottle typologies considered for each production level is reported.

The price per bottle used as a basis of comparison for the evaluation of the cost increase attributed to the application of the technologies of traceability is approximately the average price (10 € per liter) obtained by the questionnaire carried out and described in Section 2.2. This price was increased proportionally to the decrease of the bottle volume (5% more every 0.25 L reduced). This means that the price of a 0.75 L bottle is €7.88 (10.50 € L^−1^), while for 0.25 L bottles it is €2.88 (11.5 € L^−1^).

The costs attributable to the implementation of each technology was calculated considering the following elements:

- initial investment for the DAB application machine (considering 10 years amortization period);
- initial investment for the software infotracing technologies systems (ITS; considering 3 years amortization period);
- investment for the blockchain implementation (only for QR-B; considering 3 years amortization period);
- unit price of the single typology of tag;
- additional time costs for DAB application at farm level (manual application).

For the considered options, the annual depreciation costs of the machines, the ITS costs, the price of the single tag, and the additional time costs were calculated for each bottle typology (Table 3). The depreciation rate was calculated using a straight-line method [32], applying a depreciation rate of 10% and 33% respectively for application machinery and ITS, dividing the cost of the investment by the number of years, considering null the recovery value of the capital. The price of each single tag typology per production level is shown in Table 4. The different prices considering each tag typology depends on the number of tags purchased (higher the quantity, lower the price). This price, together with the DAB application machine, was obtained after a preliminary market survey.

The investment cost for the DAB application machines, the costs for ITS, and blockchain implementations are reported in Table 5.

The analysis was oriented to calculate the impact of the different technologies on the price increase of the one bottle of EVOO.

Only at farm production level, in order to highlight points of economic balance between the increase in cost due to the application of the technologies and the relative increase in price tolerated by consumers, a simulation of the increase cost for each traceability technology was carried out considering a variation of production from 5000 to 40,000 EVOO L.

## 3. Results and Discussion

### 3.1. The Consumer Questionnaire

The total respondents to the questionnaire were 1120; 1003 on the web platform and 117 by face-to-face fair interview. On the socio-demographic questions 45.4% of the respondents were females; considering the geographical residence area, 58.4% lived in Central Italy, 23.3% lived in the South of Italy, while 18.3% in the North. The composition by class of age: 19.9% of the respondents were less than 35 years old, 40.8% were 35–50 years old, and 39.3% were over 50 years old. Respondents had a high level of education: 69.0% of them were graduated [for Italy the Organization for Economic Co-operation and Development (OECD) reported 18% of graduated for the total population] [33]. The high level of education of the respondents could due to the media adopted to diffuse the questionnaire (mainly the FB CREA’s page and a specialized web magazine and FB).

Only 1.3% (15) of the respondents declared that they do not regularly consume extra virgin olive oil. Among the EVOO consumers, they were or would be willing to spend an average of 9.82 ± 4.41 euros per liter of high-quality products. A high percentage (93.9%) of the interviewed people are interested in knowing the traceability of extra virgin olive oil. Consumers were willing to pay, for the integration of traceability technologies, an additional price equal to the 17.8% with respect to the amount they commonly spent.

Considering the proposed technologies for traceability, 44.6% of consumers chose the QR-B technology, 20.9% the DAB, and 20.8% the NFC tags. Only 12.4% of the consumers did not choose any of the proposed technologies.

The QR code blockchain system appeared to be the most attractive technology from the consumer’s point of view. This is probably due from one side to its prize-winning mechanism and gamification approach [34], and from the other to the easy access to the information through QR code. Another important advantage of such a system is represented by the inalterability of traceability information input along the supply chain granted by the blockchain. Each consumer receipt of a prize (token of €0.05) for each product purchased and supply chain and production information available for the consumer and producer. Disadvantages may regard the complexity of the informatic architecture for data entry along the entire supply chain that is scarcely automatable. Instead, the DAB technology guarantees the inalterability of the bottled content and, the tag technology being based on RFID [28], the tag reading could be automatable and included in logistic platforms [35]. The main disadvantage of this technology expressed by consumers regards the complexity in obtaining information on traceability (i.e., different technologies: RFID, cash register reader, barcode, dedicated app) and the lack of an RFID antenna on normal smartphones. The NFC is a simple and widely adopted application already used for food traceability [29]. Nowadays, many smartphones have an NFC antenna integrated. Moreover, this technology provides a contact reading resulting in a poor potential automation within a logistic platform.

Mean and standard deviation values of APO and AAP with respect to the gender, age class, geographical area, and university degree is reported in Table 6. The Normality test Shapiro–Wilk verified a non-normality for the distribution of APO and AAP answer variables for all the classes of the categories Gender, Age, Geographical area, and Degree. The non-parametric Kruskal–Wallis tests (H_0_ equality of medians) showed significant differences (*p* value < 0.05) by gender and class age for both choice in favor of APO and AAP. Considering the geographical areas of residence APO revealed significant differences but not AAP together with university degree which showed non-significant differences for both APO and AAP.

These results showed males are willing to pay significantly more for APO with respect to females (10.1 vs. 9.5 €). On the contrary, females are willing to pay a significantly higher additional cost for traceability technologies (AAP) in comparison with males (19.3 vs. 16.6%). Some studies affirm that males usually are willing to spend more on foodstuff than females, but females appear to be more interested in product warranties and, for this reason, are willing to spend more on a certified product [36]. Another interesting result showed that there is a relation between APO and age classes: older people spend significantly more than younger classes. These last (with an age lower than 35) reveal an attitude to spend significantly more for technologies which granted traceability (AAP). As observed by Ascani [36], adults have increased economic possibilities due to a longer work life and a higher occupational rate, while young people are more curious in new technologies, even if they have lower economic possibilities. The survey revealed how in Northern Italian regions people spend significantly more (APO) than in Central and Southern ones for EVOO. This could be due to both the higher cost-of-living and the distance from the EVOO productive sites (in Italy mainly situated in southern regions). No differences were revealed considering the university degree. As shown in Table 7, 1120 people were interviewed, but only 1105 appeared to consume EVOO, while the remaining 15 people do not consume EVOO (rest). Among consumers only 12.4% (*n* = 139) are not interested in the proposed technologies; most of the interviewed (44.6%; *n* = 499) prefer the QR-B system, around 21% each equally preferred the other two systems (DAB and NFC).

χ^2^ tests showed non-significant (*p* value > 0.05) differences, considering the consumer’s choice for the three proposed technologies, within gender, age class, and university degree. Meanwhile, geographic area revealed significant differences; in this case, χ^2^ components showed highly significant (*p* value < 0.001) lower values for NFC in Southern Italy, QR-B in Central Italy, and DAB in Northern Italy, and highly significant (*p* value < 0.001) higher values for QR-B in Southern Italy and DAB in Central Italy.

### 3.2. Stakeholder Interviews

The three EVOO producers/suppliers were interviewed to evaluate a real degree of appreciation for each of three systems proposed, which returned interesting results. The idea of the “scratch and win” system (QR-B) was the most appreciated by the three producers/suppliers. However, just the industrial one showed real interest in its real implementation following the cost of the system retrieved on the basis of its production dimension. The DAB systems did not produce any concrete interest due to its implementation costs. Additionally, there is a sort of overlapping of such a system with the existing technologies, e.g., anti-refill cap even if this last cannot carry any information regarding the product history or traceability. The NFC systems instead appeared to be the last one in terms of appreciation due to the lack of the NFC sensors on some smartphone and of any system protection against fraud.

Food security can benefit from the technology’s transparency, relatively low transaction costs, and instantaneous applications to vehiculate the information on traceability. As reviewed by Antonucci et al. [29], generally, the robust and decentralized functionality of blockchains is used for global financial systems, but it can easily be expanded to contracts and operations such as tracking of the global food supply chain. The success of the QR-B proposed system with regard to the other ones could be mainly due to: (i. the aforementioned blockchain transparency aspects; ii. the use of incentives, such as financial rewards (expressed as token in the proposed QR-B system) mechanism [37], and iii. the gamification strategy also obtained with the proposed “scratch and win” system). A variety of business sectors have been buffeted by the diffusion of mobile technology, a trend that presents a variety of difficult challenges but interesting opportunities to marketers. One such opportunity is gamification, which is enhancing appeal to mobile consumers [38]. Gamification may be distinguished from traditional loyalty programs by providing added social and motivational benefits through product usage rather than only expenditures [37,39]. Mobile gamification is especially useful to reach consumers in phone-centric parts of the world and millennials [40]. Gamification executed on the mobile platform has the potential to affect an important set of retailing outcomes, to entertain customers, to accelerate repurchase, to retain customers, and to contribute to in-store engagement. In fact, its effect might be felt throughout the consumer decision process.

### 3.3. Economic Analysis

**Figure 1:**
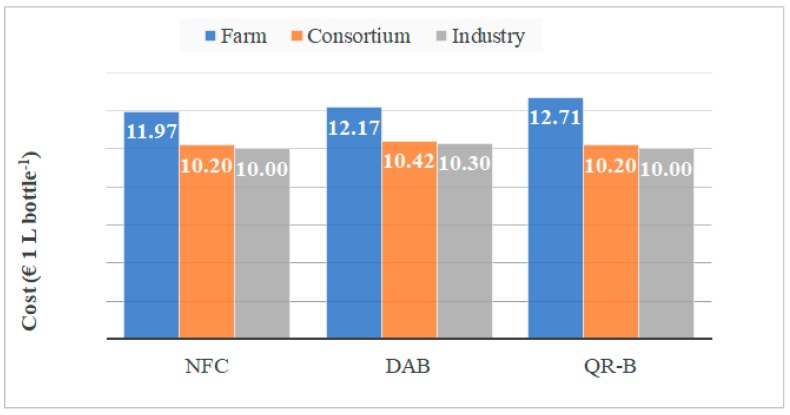
Final cost of the bottle (1 L) including additional costs due to the application of the three technologies for the three production levels (initial price equal to 10 € bottle^−1^). NFC: Near Field Communication; DAB: Dispositivo Antimanomissione per Bottiglie; QR-B: Quick Response Blockchain.

The results of the economic analysis, referring to the cost increase for the application of the three different traceability technologies and in relation to the three different typologies considered (0.25, 0.75, and 1 L), are reported in Table 8. Generally speaking, for all the technological systems, the impact on the baseline price is always greater for the farm level production, while it decreases considerably as the application level increases. This is due to the better level of amortization of the capital invested and lower tag purchase costs for higher productions. In particular, the highest increase in cost (+31%) was obtained for 0.25 L bottle in the DAB system, at farm production level, while the lowest (minor then 0.05%) was registered for all bottle typologies at industrial production level for NFC and QR-B systems. Figure 1 shows the final cost of a 1 L bottle, for the different scenarios examined. The highest price is found for the QR-B system, in the case of farm production level, with 12.71 € L^−1^, while the NFC and QR-B systems, obtained the lowest prices, corresponding to 10.20 and 10.00 € L^−1^ respectively for consortium and industry level. For these two levels, the prices of DAB system increase respectively to 10.42 and 10.20 € L^−1^.

**Figure 2:**
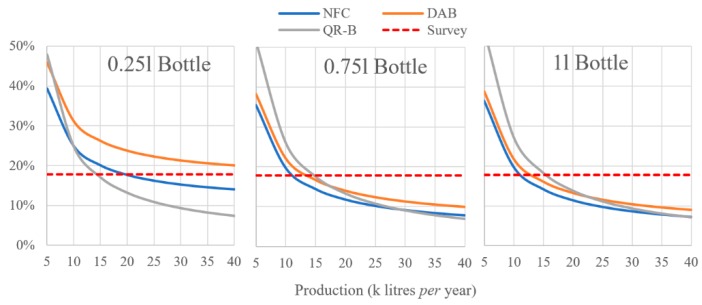
Variation of the increase in cost (in %) per bottle, at farm level only, for three systems in relation to the annual oil production.

Considering that only at the level of farm production the percentage of cost increase, compared to the baseline price, is more significant, the differences between the three systems, for this production level are highlighted in Figure 2. The simulation was carried out in consideration of a production interval between 5000 and 40,000 L per year. The curves are compared with the percentage of cost increase that respondents at the questionnaire are willing to pay (17.8%; horizontal dashed line in Figure 2). For 0.25 L bottles, the DAB system appears always to be above 17.8%, while, for QR-B and NFC, the break-even point would be obtained to 15,000 and 20,000 L of production respectively. For 0.75 and 1 L bottles, this point is reached by the three systems at a production level below 15,000 L, with the DAB system that recovers compared to QR-B.

**Figure 3:**
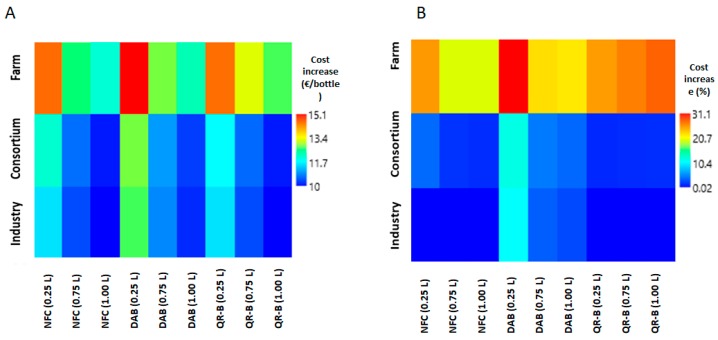
(**A**) Matrix plot of the cost increase per bottle in relation to the technology used and the production level. (**B**) Matrix plot of the cost increase, in percentage, in relation to the technology used and the production level.

For a visual comparison among the different scenarios, Figure 3A,B shows the price increase in absolute value and as a percentage of the unit cost of the EVOO with respect to a baseline price of 10 € L^−1^.

The economic analysis demonstrated how the proposed technologies able to vehiculate EVOO information on traceability are ready for a real application from a sustainable economic point of view for a consortium (mid) and industrial (high) production level. The key aspect concerns the methodological approaches to be applied to obtain information on EVOO geographical traceability. Nowadays, these methods are mainly based on chromatographic, spectroscopic, and isotopic techniques [41]. The results obtained by the survey revealed that among Italian consumer respondents, the traceability information vehiculated with smart technologies are highly welcome. Consumers are not only ready to accept but also ready to spend a high extra-budget for the additional information if they are true (i.e., the concept of trust) [42]. The food market is, in general, a rich market with respect to other sectors. A similar questionnaire has been proposed in the wood sector [43], returning a lower willingness to pay result (around 2.5% for the technological implementation).

In previous years, stronger needs to provide new advances within the agri-food sector leading to improve efficiency and versatility of the actual traceability systems were underlined [17]. Some examples regard the use of blockchain for food supply chain transparency and for anti-fraud systems. As reported by Antonucci et al. [29] the blockchain technology was used to ensure the quality of coffee products and their chain production associating all the information on a univocal QR Code, meanwhile a Milk Verification Project prototype was developed to tackle food fraud. Another example of new agri-food traceability system was reported by Trebar et al. [44] using RFID data loggers for the fresh fish supply chain in acquiring non-stop temperature during the logistics process of warehousing of fish and the transport from cold store to the retail or private consumers.

## 4. Conclusions

This study contributes to the EVOO production panorama providing concrete tools for improving the internal logistics of the company on the one hand and promoting infotracing and safeguarding consumers on the other. Even if the technologies have been present on the market for some years, now they are almost new to the olive oil production food chain.

EVOO represents the one of the excellences of Italian products; it is a basic ingredient of the Mediterranean diet; for this reason, it must be protected from fraud and sophistication that could damage “made in Italy” and have repercussions both for producers and consumers. This study showed some interesting and surprising outcomes. Following the questionnaire results, the high level of the respondent education could mainly due to the media (a specialized online tool) adopted to spread the questionnaire. First of all, the unexpectedly high willingness to pay (+17.8%) by Italian consumers for the implementation of traceability information on EVOO mediated by smart technologies. This interesting result is not affected by the high level of education of the respondents (63% graduated) even if it may have influenced the considerable percentage (93.9%) of those interviewed. However, the education level and technological knowledge may not be properly on par. Furthermore, according with the survey results, the age composition revealed different spending behavior patterns. Respondents less than 35 years old (19.9% of the respondents) were less available to pay additional costs for the implementation of information regarding traceability. A future study could try to improve the effectiveness of the present work crossing data related to technological knowledge along with those already asked.

Although blockchains are not yet very widespread in EVOO traceability, their use may be an excellent solution to ensure reliability, transparency, and security, especially for those commodities susceptible to fraud such as EVOO. Among the three proposed technologies for traceability, consumers greatly prefer the QR-B system, despite the different advantages linkable to the other systems. The success of the QR-B could be ascribable to different aspects: the indubitable cutting-edge fashion trend of blockchain in the food sector, the use of incentives (financial rewards mechanism), the easy-to-use QR-code, and the gamification strategy. Another interesting result regards the economic analysis which reports a sustainable implementation of the three technologies proposed starting from a mid-production EVOO level. However, it must be said that further studies should investigate the deeper costs that farms/industries etc., would have to pay in relation to the business planning, prior the technological system adoption.

On the basis of the obtained results, it is possible to predict an evolution of the proposed traceability systems merging the benefits of each technology into a syncretic one, aiming to preserve the bottle content (DAB), the easy-to-use properties (NFC and QR-code) with the QR-B aforementioned qualities, and integrating other information, such as the preservation status from bottling to consumers adopting functional inks.

## Figures and Tables

**Table 1:** Electronic labels used for the questionnaire.

Electronic Label	Description	Reader Device	RFID	NFC	Barcode	Blockchain
NFC	Label placed underneath the external label and activated via a smartphone antenna to read traceability information	Smartphone		X		
DAB	Tamper-proof device composed of a bottle cap, containing a RFID adhesive label, which externally covers the traditional one	Dedicated antenna device	X			
QR-B	QR code tag printed on the external label and protected by a “scratch and win” system to allow the consumer to obtain the reward implemented with blockchains	Smartphone			2D QR-code	X

NFC: Near Field Communication; DAB: Dispositivo Antimanomissione per Bottiglie; RFID: Radio Frequency Identification; QR-B: Quick Response Blockchain. The “X” identifies the presence of the selected device.

**Table 2:** Questionnaire format implemented in Microsoft Forms.

Index	Questions	Label	Potential Answers
Q1	Which is your gender?	Gender	Female; Male
Q2	Which is your age class?	Age class	<35; 36–50; >50
Q3	Which is your Italian geographical area of residence?	Geographical area	North; Central; South
Q4	Do you have a scholar degree?	Degree	Yes; No
Q5	Do you usually consume EVOO?		Yes; No
Q6	How much do you spend to buy a bottle of one liter of EVOO?	APO	€ L^−1^
Q7	Are you interested in knowing the exact provenance of the EVOO that you buy?		Yes; No
Q8	Which is the additional price that you willing to pay to purchase one bottle of EVOO (1 L) with a traceability technology integrated?	Used to derive AAP	€ L^−1^
Q9	Which of these traceability systems would you buy?		NFC; DAB; QR-B

EVOO: extra virgin olive oil; APO: high quality extra virgin olive oil purchased on average; AAP: acceptable additional cost to pay.

**Table 3:** Production of bottles (number per year) considered in relation to three production levels.

Production Level	Bottle Typology (*n*/year)		
	0.25 L	0.75 L	1 L
Farm	40,000	13,333	10,000
Consortium	800,000	266,667	200,000
Industry	120,000,000	40,000,000	30,000,000

**Table 4:** Market prices of the different tags considered for the three production levels (in € unit^−1^).

Production Level	NFC	DAB	QR-B
Farm	0.3000	0.3500	0.0450
Consortium	0.1000	0.3000	0.0010
Industrial	0.0100	0.2000	0.0001

**Table 5:** Investment cost of the Dispositivo Antimanomissione per Bottiglie (DAB) application machine, software for the infotracing technologies systems (ITS) and blockchain costs in relation to the three production levels (in €).

Production Level	DAB Application	ITS	Blockchain
Farm	3000	50,000	30,000
Consortium	44,500	60,000	60,000
Industrial	539,500	80,000	180,000

**Table 6:** Average values and standard deviations of mean purchase for high quality extra virgin olive oil (EVOO) (APO) and acceptable additional cost to pay for traceability technologies (AAP) with respect to gender, age class, geographical area (Italy), and university degree.

	*n*	APO (€/L)	AAP (%)
Gender			
F	508	9.5 ± 4 ^a^	19.3 ± 19.4 ^a^
M	612	10.1 ± 4.7 ^b^	16.6 ± 16.4 ^b^
Age			
<35	223	9.4 ± 4.8 ^a^	23 ± 21.8 ^a^
36-50	456	9.6 ± 4.2 ^a^	16.6 ± 16.4 ^b^
>50	441	10.2 ± 4.4 ^b^	16.4 ± 16.6 ^b^
Geographical area			
North	205	10.8 ± 4.6 ^a^	17 ± 16.7 ^a^
Central	654	10 ± 4.3 ^b^	17.4 ± 17.1 ^a^
South	261	8.6 ± 4.3 ^c^	19.5 ± 20.2 ^a^
Degree			
No	347	9.7 ± 4.8 ^a^	19 ± 19.8 ^a^
Yes	772	9.9 ± 4.2 ^a^	17.3 ± 16.9 ^a^

Differences between medians that share a letter are not statistically significant (Kruskal–Wallis test).

**Table 7:** The consumer’s choice on the three proposed technologies (Near Field Communication (NFC), Dispositivo Antimanomissione per Bottiglie (DAB), and Quick Response Blockchain (QR-B)). “Rest” indicates people that do not consume extra virgin olive oil (EVOO).

	*n*	%
NFC	233	20.8
QR-B	499	44.6
DAB	234	20.9
None	139	12.4
Rest	15	1.3

**Table 8:** Results of the economic analysis related to different scenarios examined.

**Total Cost (€/Bottle)**									
**Level**	**NFC**			**DAB**			**QR-B**		
	**(0.25 L)**	**(0.75 L)**	**(1 L)**	**(0.25 L)**	**(0.75 L)**	**(1 L)**	**(0.25 L)**	**(0.75 L)**	**(1 L)**
Farm	3.59	9.43	11.97	3.77	9.62	12.17	3.59	9.92	12.71
Consortium	3.00	8.05	10.20	3.21	8.27	10.42	2.93	8.03	10.20
Industry	2.88	7.88	10.00	3.18	8.18	10.30	2.88	7.88	10.00
**Cost Increase (%)**									
**Level**	**NFC**			**DAB**			**QR-B**		
	**(0.25 L)**	**(0.75 L)**	**(1 L)**	**(0.25 L)**	**(0.75 L)**	**(1 L)**	**(0.25 L)**	**(0.75 L)**	**(1 L)**
Farm	24.93	19.68	19.67	31.10	22.13	21.67	24.75	25.97	27.12
Consortium	4.35	2.22	2.00	11.50	4.97	4.22	1.77	1.92	2.01
Industry	0.04	0.02	0.02	10.46	3.84	3.03	0.03	0.03	0.03
